# Atomoxetine-Induced Pheochromocytoma and Paraganglioma Crisis Managed With Veno-Arterial Extracorporeal Membrane Oxygenation

**DOI:** 10.7759/cureus.73582

**Published:** 2024-11-13

**Authors:** Hirotsugu Kaneshima, Naoya Miura, Asuka Tsuchiya, Seiji Morita, Yoshihide Nakagawa

**Affiliations:** 1 Department of Emergency and Critical Care Medicine, Tokai University School of Medicine, Isehara, JPN

**Keywords:** atomoxetine, attention deficit hyperactivity disorder, cardiac arrest, extracorporeal membrane oxygenation, pheochromocytomas and paragangliomas

## Abstract

Pheochromocytoma and paragangliomas (PPGLs) crises can be triggered by various factors, including norepinephrine reuptake inhibitors used to treat attention deficit hyperactivity disorder (ADHD), which worsen symptoms in patients with PPGLs. Therefore, attention should be paid to the potential for serious adverse reactions in patients with PPGLs taking ADHD medications. A 21-year-old man presented to the emergency department with acute onset of severe respiratory and circulatory failure after initiating atomoxetine treatment. During preparation for hospital admission, his respiratory and circulatory status deteriorated, requiring emergency intubation and mechanical ventilation and transfer to our institution for further evaluation and treatment. Profuse sweating and sinus tachycardia were observed, and echocardiography revealed a significantly reduced ejection fraction. Contrast-enhanced computed tomography of the trunk revealed a 50 mm tumour anterior to the inferior vena cava and a 20 mm enhancing tumour in the left adrenal gland. Treatment with the α-blocker, phentolamine, was initiated on the grounds of cardiogenic shock induced by an endocrine disorder such as PPGLs. However, the patient developed bradycardia and hypotension, progressing to pulseless electrical activity (PEA), for which cardiopulmonary resuscitation (CPR) was initiated. After the administration of adrenaline (1 mg), a return of spontaneous circulation was achieved. Veno-arterial extracorporeal membrane oxygenation (VA-ECMO) was initiated to prevent further cardiac arrest. An intra-aortic balloon pump (IABP) was inserted to reduce the cardiac workload. Circulatory dynamics gradually stabilised, and treatment with VA-ECMO was discontinued on day 4 after admission as the ejection fraction improved to approximately 50%. On day 6, the patient was successfully extubated, respiratory support was discontinued, and he was discharged on day 25, confirming the diagnosis of PPGLs with no evidence of higher brain dysfunction. Outpatient management included dose adjustment of the α1-blocker, and he was readmitted for surgical removal of the tumour. The postoperative course was uneventful, with a notable improvement in ADHD symptoms. This case report highlights the importance of a multidisciplinary approach for the diagnosis and management of patients with symptoms suggesting psychiatric or endocrine disorders.

## Introduction

Pheochromocytomas and paragangliomas (PPGLs) are catecholamine-producing tumours originating from chromaffin cells in the adrenal medulla or paraganglionic system that present with various symptoms such as hypertension, palpitations, tachycardia, headache, and feelings of anxiety [1,2]. PPGLs-crisis can be precipitated by various triggers, including food intake, urination, tumour removal, and the use of medications, such as beta-blockers, high doses of dexamethasone, glucagon, contrast agents, metoclopramide, and tricyclic antidepressants [3-6]. Noradrenaline reuptake inhibitors used in attention deficit hyperactivity disorder (ADHD) treatment [7] are contraindicated in patients with PPGLs because of the potential symptom exacerbation [8].

Here, we report the case of a patient with PPGLs who developed cardiac arrest after taking ADHD medication and was subsequently saved by the initiation of veno-arterial extracorporeal membrane oxygenation (VA-ECMO). This case highlights the importance of recognising the potential for severe adverse reactions in patients with PPGLs taking ADHD medications.

## Case presentation

**Figure 1:**
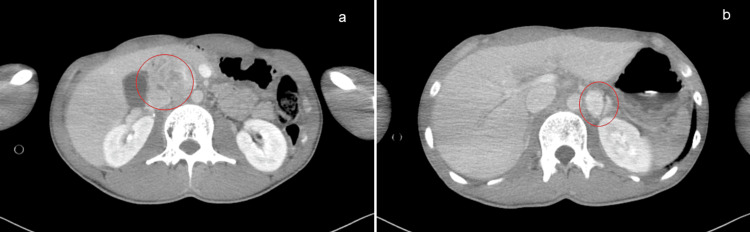
Axial CT with intravenous contrast Axial CT with intravenous contrast demonstrate a 50 mm tumour anterior to the inferior vena cava (a) and a 20 mm enhancing tumour in the left adrenal gland (b).

A 21-year-old Japanese male with no previous medical history presented to the emergency department with acute-onset severe respiratory and circulatory failure. He was started on atomoxetine, which is approved for ADHD treatment, and was diagnosed with excessive daytime sleepiness. Following the initiation of treatment, the patient experienced nausea, which he believed to be a side effect, and continued the medication. One week after starting the medication, the nausea and abdominal pain became so severe that he presented to the emergency department of the hospital. He had hypoxaemia, and computed tomography (CT) revealed decreased lung transparency and a retroperitoneal mass. While preparing for hospital admission for further investigation, his respiratory and circulatory status deteriorated, necessitating emergency intubation and mechanical ventilation, along with the initiation of noradrenaline for systemic management and transfer to our facility for further evaluation and treatment. Upon arrival at our hospital, the patient's vital signs were as follows: Glasgow Coma Scale (GCS), 1-T-1; temperature, 39.0 °C; blood pressure, 104/62 mmHg; heart rate, 158 beats per minute; respiratory rate, 30 breaths per minute; and SpO2, 92% at 15 L/min of oxygen via a reservoir mask. Metabolic acidosis with acidemia (pH 7.23) and lactic acidosis (4.9 mmol/L) were observed. Profuse sweating and sinus tachycardia were noted, and echocardiography showed a significantly reduced ejection fraction (EF) of 10-20% (Video 1). Contrast-enhanced CT of the trunk identified a 50 mm tumour anterior to the inferior vena cava and a 20 mm enhancing tumour in the left adrenal gland (Figure 1).

**Video 1:** Post-admission echocardiogram This echocardiogram was performed after the initiation of VA-ECMO treatment and, therefore, reflects the patient's cardiac function following some degree of improvement.

**Figure 2:**
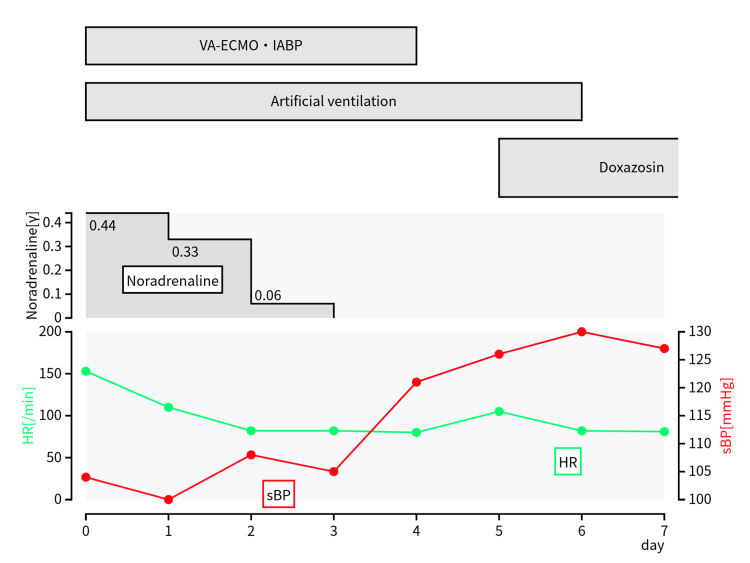
Progress chart after admission

Considering cardiogenic shock induced by an endocrine disorder such as PPGL, treatment with the α-blocker phentolamine was initiated. However, the patient soon developed bradycardia and hypotension, which progressed to pulseless electrical activity (PEA), for which cardiopulmonary resuscitation (CPR) was initiated. After the administration of adrenaline (1 mg), the return of spontaneous circulation was observed. The VA-ECMO was initiated to prevent further cardiac arrest. An intra-aortic balloon pump (IABP) was inserted to reduce the cardiac workload, and the patient was admitted to our intensive care unit for targeted temperature management. Circulatory dynamics gradually stabilised, and treatment with VA-ECMO was completed on day 4 after admission as the EF improved to approximately 50%. On day 6, the patient was successfully extubated, and respiratory support was discontinued (Figure 2).

**Figure 3:**
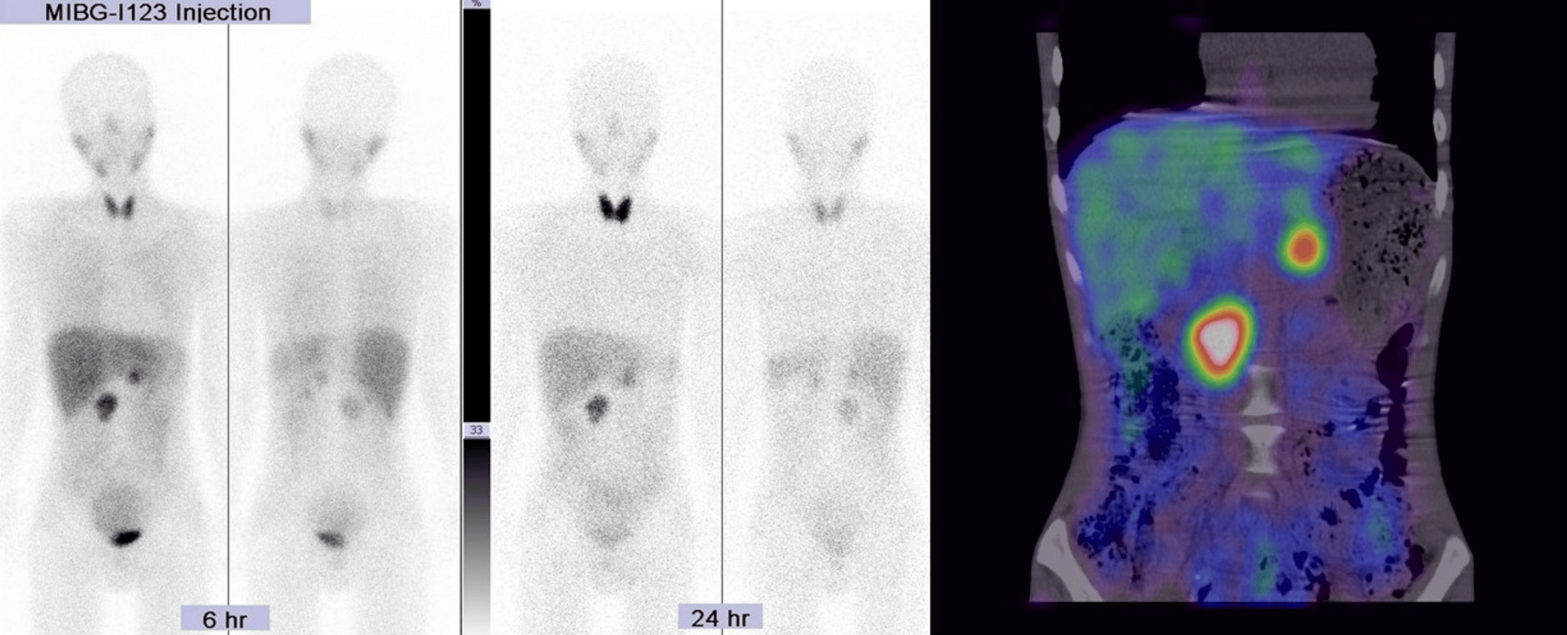
123I-MIBG scintigraphy showed uptake in the tumour

Concurrently, administration of oral α-1 blocker was initiated and transferred to the general ward on day 8. The concentration of urinary catecholamines was more than three times the upper limit of the standard value, and 123I-MIBG scintigraphy revealed uptake in the tumour (Figure 3), confirming the diagnosis of PPGLs.

With the ability to ambulate independently on day 25 without any signs of higher brain dysfunction, the patient was discharged. Outpatient management included dose adjustment of the α1-blocker, and he was readmitted for surgical removal of the tumour of the left adrenal and the anterior to the inferior vena cava. The patient’s postoperative course was uneventful, with a notable improvement in ADHD symptoms.

## Discussion

Investigation of the epidemiology of PPGLs indicated that hypertensive- or PPGL-crisis can lead to serious life-threatening cardiac and cerebrovascular complications [4,6,9,10]. Catecholamine-induced cardiomyopathy and refractory cardiogenic shock may also occur. The pathophysiology of cardiogenic shock following PPGLs-crisis is not fully understood [11,12]. Proposed mechanisms include myocardial stunning, coronary vasospasms, and cellular toxicity [13]. Our case provides several critical insights into the management and implications of PPGLs, particularly when using medications for ADHD complicated by cardiogenic shock. First, our experience strongly suggests that in cases of PPGL-induced crises leading to shock or cardiac arrest, prompt initiation of VA-ECMO should be considered. The favourable prognosis associated with early VA-ECMO intervention, as evidenced by improved cardiac function and a high in-hospital survival rate of 87% in a compilation of 62 cases [14], reinforces its significance as an appropriate mechanical circulatory support in emergency settings.

Second, the overlapping symptomatologies of PPGLs and ADHD present diagnostic challenges. The manifestation of psychiatric symptoms such as anxiety and panic disorders in PPGLs is well documented [15]. Interestingly, a review of patients diagnosed with pheochromocytoma at the National Institutes of Health between 2006 and 2014 revealed that 21% of patients aged <18 years were previously diagnosed with ADHD [16]. Notably, in three of these cases, ADHD-like symptoms resolved following the surgical removal of the tumour. This finding suggests a potential misattribution of symptoms to psychiatric conditions when an underlying endocrine disorder may be the root cause.

Lastly, the exacerbation of PPGL symptoms following the administration of atomoxetine, a norepinephrine reuptake inhibitor used in the treatment of ADHD, highlights the critical importance of considering PPGLs as a differential diagnosis before prescribing such medications. Atomoxetine increases serum norepinephrine levels and can significantly worsen the symptoms of PPGLs, potentially leading to life-threatening hypertensive crises [17]. This case illustrates the complex interplay between pharmacotherapy for ADHD and the management of PPGLs, emphasising the need for thorough screening of PPGLs before initiating treatment with norepinephrine reuptake inhibitors.

## Conclusions

In conclusion, this case report not only illustrates the acute management challenges posed by PPGLs but also highlights the broader implications for the differential diagnosis of ADHD and the careful consideration required when prescribing medications that may exacerbate this condition. This underscores the importance of an interdisciplinary approach in the diagnosis and management of patients presenting with symptoms indicative of either psychiatric or endocrine disorders.
